# The Second Law and Entropy Misconceptions Demystified

**DOI:** 10.3390/e22060648

**Published:** 2020-06-11

**Authors:** Milivoje M. Kostic

**Affiliations:** Department of Mechanical Engineering, Northern Illinois University, DeKalb, IL 60115, USA; kostic@niu.edu

**Keywords:** entropy, Second Law of thermodynamics, Second Law challenges, Second Law misconceptions

## Abstract

The challenges and claims of hypothetical violations of the Second Law of thermodynamics have been a topic of many scientific, philosophical and social publications, even in the most prestigious scientific journals. Fascination with challenging the Second Law has further accelerated throughout the development of statistical and quantum physics, and information theory. It is phenomenologically reasoned here that non-equilibrium, useful work-energy potential is always dissipated to heat, and thus thermodynamic entropy (a measure of thermal disorder, not any other disorder) is generated always and everywhere, at any scale without exception, including life processes, open systems, micro-fluctuations, gravity or entanglement. Furthermore, entropy cannot be destroyed by any means at any scale (entropy is conserved in ideal, reversible processes and irreversibly generated in real processes), and thus, entropy cannot overall decrease, but only overall increase. Creation of ordered structures or live species always dissipate useful energy and generate entropy, without exception, and thus without Second Law violation. Entropy destruction would imply spontaneous increase in non-equilibrium, with mass-energy flux displacement against cause-and-effect, natural forces, as well as negate the reversible existence of the very equilibrium. In fact, all resolved challengers’ paradoxes and misleading violations of the Second Law to date have been resolved in favor of the Second Law and never against. We are still to witness a single, still open Second Law violation, to be confirmed.

## 1. Introduction

In addition to language semantics, there are many perplexing issues related to the very essence of thermodynamics, especially the Second Law fundamentals, its subtle definitions and ambiguous meanings of key concepts, including the nature of heat and entropy [1,2]. The following essentials are vitally critical since they encompass, irrespective of the system and process details, all space and time scales: (1) the “*reversible equivalency*” implies that if the Second Law can be violated in any unique way, then it would be universally violated, and (2) forcing is directional and “*forced directionality*” imply irreversibility, i.e., process impossibility in the opposite direction. It is hoped that this treatise will also help demystify some recent challenges of the Second Law of thermodynamics and promote constructive future debates.

The Second Law made its appearance around 1850, and almost a century later, the physicist and philosopher Bridgman [3] still complained that “there are almost as many formulations of the Second Law as there have been discussions of it.” Even today, the Second Law remains so obscure, due to the lack of its comprehension, that it continues to attract new efforts at clarification, including by this author [4,5,6,7,8]. Einstein, whose early writings were related to the Second Law, remained convinced throughout his life that “thermodynamics is the only universal physical theory that will never be refuted”. Namely, the phenomenological laws of thermodynamics have much wider, including philosophical, significance and implication than their simple expressions based on the experimental observations.

The current frenzy about the Second Law violation (the perpetual motion machine of the second kind, PMM2, obtaining useful energy from within equilibrium, or spontaneous creation of non-equilibrium work potential and entropy destruction) is in many ways similar to the prior frenzy about the First Law violation (the perpetual motion machine of the first kind, PMM1, obtaining energy from nowhere). Related position had been expressed by Eddington’s well-known statement [9]: “The law that entropy always increases—the Second Law of thermodynamics—holds, I think, the supreme position among the Laws of Nature.” Sometimes, even highly accomplished scientists in their fields do not fully comprehend the essence of the Second Law of thermodynamics. It is hard to believe that a serious scientist nowadays, who truly comprehends the Second Law and its essence, would challenge it based on incomplete and elusive facts.

As the fundamental laws of nature and thermodynamics are expanded from simple systems in physics and chemistry to different space and time scales and to much more complex systems in biology, life and intelligent processes, there are more challenges to be comprehended and understood. Thermodynamic entropy should be further reasoned, refined, and explained for what it is, and not be misrepresented as something it might be or it is not [6].

Phenomenological thermodynamics has supremacy over other disciplines, due to its logical reasoning based on the fundamental cause-and-effect principle and without regard to the system’s complex dynamic structure, and even more complex related interactions. The fundamental physical laws are independent from any system structure, and they should take primacy over any special analysis and elaborate simulations based on hypothetical approximations and limitations of modeling of systems, their properties and processes. 

More importantly, the micro- and submicro-simulations and experimental analyses are also based on the fundamental laws, and therefore, they cannot be used to prove or worse to negate those fundamental laws. 

Afterall, the science and technology have evolved over time on many scales and levels, so that we now have the advantage to look at its historical developments more comprehensively and objectively than the pioneers [1,2,10,11]. Therefore, on purpose, only phenomenological reasoning without any mathematics is presented here. As Anthony Legget, a Nobel laureate, recently commented [12], emphasizing “Mathematical convenience versus physical insight … that theorists are far too fond of fancy formalisms which are mathematically streamlined but whose connection with physics is at best at several removes … and he hurtfully agreed with Philippe Nozieres that ‘only simple qualitative arguments can reveal the fundamental physics’.”

The intention of this treatise is not to review the vast *entropy* and *Second Law* literature, but to present this author’s long-contemplated reflections on the essence of the Second Law and the physical meaning of entropy, and to put certain physical and philosophical concepts in historical and contemporary perspective. Therefore, only selected seminal publications and related publications by this author are referenced.

Regardless of never-ending obstacles and controversies, but due to many creative and some mystical writings, mostly scattered throughout diverse literature, the motivation and inspiration for challenging the Second Law of thermodynamics are still flourishing. Fascination about challenging the universality of the Second Law has accelerated throughout the development of statistical and quantum physics, and information theory [13]. However, the vast majority of scientific community up to now have not been convinced with the Second Law challengers and demonologists.

## 2. The Essence of “Mass-Energy, Entropy and the Second Law Fundamentals” 

Nature, our universe, is made up of mass-energy in non-equilibrium, consisting of non-uniform mass-energy structures exhibiting a spontaneous (self-driven) forced tendency to interact and re-displace mass-energy towards uniform equilibrium(Box 1). During such interactions (forced processes), mass-energy is conserved (the First Law), and such forced processes are taking place in a certain irreversible direction, from higher to lower mass-energy density potential, towards mutual equilibrium, and not spontaneously in the reverse direction, not opposite to the forced tendency direction (the Second Law). Non-equilibrium mass-energy (with reference to mutual equilibrium) represents the useful work-energy potential (from now on, “*work potential*” for short) available as the driving force for displacing/rearranging existing structures into new structures (desirable or not). Such work potential may be ideally conserved in reversible processes but will inevitably and irreversibly dissipate, in time and space, into thermal heat and generate entropy (increase thermal space), until irreversible equilibrium is achieved. 

The existing non-equilibrium work potential may only be transferred (re-arranged) and/or dissipated (converted to thermal energy), but new work potential cannot be spontaneously generated from within equilibrium nor otherwise (the Second Law). Mass-energy equilibrium, or more accurately quasi-equilibrium, may also be achieved within certain, bounded mass-energy stable structures while still having a self-sustained, non-uniform mass-energy concentration (non-uniform mass-energy density). If such bounds are broken by forcing (on expense of external work potential, or from within work potential, e.g., fuel ignited in heat engine), further processes may take place and lead to a new stable equilibrium until overall equilibrium is achieved with net-zero work potential (thermal death).

Box 1 **Natural Processes and Laws.**

All processes in nature are irreversibly driven in a forced direction by non-equilibrium useful energy, where mass-energy flux is transferred while conserved (the First Law), but in part (and ultimately in whole at equilibrium), the useful energy is dissipated to thermal heat, and thus always and irreversibly generating entropy (the Second Law).

In general (without exception, for all space and time scales), mass-energy is conserved (the First Law), while available work potential (as cause of all forced processes) is irreversibly dissipated to heat, and thus always generating entropy. Therefore, without exception, neither mass-energy, nor available work potential, can be generated, nor can entropy be destroyed, but entropy is always, irreversibly generated due to work potential dissipation to heat (Box 2).

The fundamental terminology in thermodynamics has very specific meaning, distinct from common etymology. For example, “reduction in entropy” often means *complete* or *total* or *overall* entropy reduction, involving all interacting systems (including interacting near-surroundings, as if they are isolated from the far-surroundings). Of course, we could reduce the entropy of any system by transferring it out to other systems, but we cannot “completely reduce” the entropy of all interacting systems, since there is no way to destroy entropy locally nor temporarily—entropy is always generated (i.e., overall increased). Likewise, we could reverse any process by forcing from outside, but a *reversible process* in thermodynamics means *completely reversible* or *overall reversible*—that is, to reverse all previously interacting systems (spontaneously from within) without any *trade-off* forced-interactions with other external systems. For closed and isolated systems, the terminology is more obvious, but for open and more complex systems, the fundamental laws are equally valid if all interacting systems and processes are properly accounted for. Afterall, all systems in nature are open and all processes are irreversible, while a closed system and reversible process are only useful idealizations, as are rigid or ideally elastic bodies, adiabatic boundaries, stationary processes, or perfect equilibriums, etc.

Box 2 **It Is Hard to Believe Nowadays.**

Sometimes, even highly accomplished scientists in their fields do not fully comprehend the essence of the Second Law of thermodynamics.

*     It is hard to believe that a serious scientist nowadays, who truly comprehends the Second Law and its essence, would challenge it based on incomplete and elusive facts.*

Real thermodynamic *entropy* is defined as thermal energy displacement, i.e., as an integral ratio of thermal heat and related absolute temperature (entropy is precisely quantified and tabulated in reference data tables). Therefore, entropy is a macro-thermal property related to random thermal motion, i.e., “thermal disorder”, and not related to any other disorder [6]. The fundamental Second Law of thermodynamics describes all natural processes where energy is transferred and/or converted due to a natural tendency to spontaneously force mass-energy displacement (redistribution) from higher to lower energy concentration (energy intensive-potential), approaching mass-energy equilibrium (*equipartition* of mass-energy), and thus defining the meaning of *forcing directionality* and *irreversibility* of all natural processes, and impossibility otherwise. Therefore, natural processes (spontaneous forcing of mass-energy displacement, not only thermal, but in general) cannot go spontaneously in opposite to the natural forcing direction. That is, spontaneous heat transfer must go from higher towards lower temperature (thermal potential), mass has to free fall or free flow (spontaneously) from higher to lower elevation (gravitational potential; downwards, not upwards), space expansion from higher to lower pressure, electricity flows from higher to lower electrical potential (voltage), species disperse from higher to lower concentrations (chemical potential), etc. The essence here is “spontaneously” meaning in self-driven forcing direction and not in the opposite or reverse direction (irreversible thermodynamic-spontaneity). Of cause, we may “externally force” (non-spontaneously, at the expense of external non-equilibrium, or from within quasi-equilibrium) by using special devices and processes (including technical, intelligent or life processes), to “trick” heat to net transfer (or net transport) from lower to higher ambient temperature (like in refrigeration), or lift mas or pump fluid to higher elevation, or transport electricity to higher voltage, or concentrate species, or build new-ordered structures, etc. However, all such processes will dissipate work potential and generate heat and entropy (only in ideal limit conserve entropy), and they will not violate the Second Law (will not destroy entropy), even though they may appear to be magically displacing mass-energy against natural forces, i.e., against spontaneity. 

Even more elusive are life processes or processes at small-space and small-time scales (like quantum, subatomic and molecular fluctuations) due to the limitations of our observation and comprehension, where lack of proper accounting of all forced interactions and incomplete definitions of entropy are more subtle and may lead to misleading conclusions. 

## 3. The Three Essential “Entropy and Second Law Misconceptions”

### 3.1. Is Entropy a Measure of Any Disorder?

Thermodynamic *entropy* is the thermal displacement (“thermal motion space”, i.e., thermal disorder displacement space). Entropy is *not* any space disorder, nor form, nor functional disorder. Real thermodynamic entropy is related to thermal energy and its (heat) transfer only (Box 3). Expanding entropy to any type of disorder or information is misleading and a source of many misconceptions, since those disorders are mutually independent concepts. Entropy is a thermal property, not a statistical concept, although it may be described with statistical and probabilistic methods. The modern generalization that “*Entropy is a measure of [any] disorder or randomness in the system...*” is too general and over-reaching, based on inadequate and misleading analogy, and thus inappropriate [6].

Box 3 **Entropy Is Thermal Randomness, Not Any Randomness.**

    Real thermodynamic entropy is the thermal displacement space, related to thermal motion randomness, and not related to any other randomness. Entropy is certainly a thermal concept and not a statistical nor probabilistic concept *per se*, just because statistical modeling is used to describe it.

The modern generalization that *“Entropy is a measure of [any] disorder or randomness in the system...”* is too general and overreaching, based on inadequate and misleading analogy, and thus inappropriate.

To restate it again: real thermodynamic entropy is the thermal displacement space, related to thermal motion randomness, and not related to all other randomnesses. Entropy is a physical macro-property and certainly not a statistical and probabilistic concept *per se*, just because statistical modeling is used to describe it. Granted, there are certain benefits of simplified statistical descriptions to better comprehend the randomness of thermal motion and related physical quantities, but the limitations should be stated so the generalizations are not over-stretched and the real physics overlooked, or worse discredited.

Natural phenomena are not subordinate to our science laws, but the other way around—our science laws only model and describe natural phenomena including unavoidable simplifications and limitations. Therefore, *entropy* is a measure of *thermal* disorder or *thermal* randomness in a system. For simple systems, such as ideal gasses, entropy may be well described by statistical/probabilistic modeling of thermal particle motion, but it is inappropriate to expand it for all other possible disorders and generalize it into a “*monster entropy*”.

Entropy, as thermal disorder, is always generated (produced), in all processes without exception, and cannot be destroyed (no “thermal order”) by any means. This should not be confused with local entropy change that could increase or decrease due to entropy transfer. However, the other orders or disorders, such as structural (subatomic, atomic, molecular, bulk), form or functional (structures and devices of all kinds), information, intelligence, life, social, or philosophical, can be created or destroyed by physical processes, and are always accompanied with entropy generation. This demonstrates that all other orders and disorders are autonomous concepts and independent from thermal energy disorder, the latter quantified by thermodynamic entropy. Other types of quasi-entropies can be defined to quantify other disorders, but they should not be confused with classical, thermodynamic entropy. 

Expanding the classical concept of thermodynamic entropy (thermal energy disorder) to abstract or general concepts (for any and all types of disorders) or information is ‘overreaching’ and can be a source of many inconsistencies and misconceptions. A system form and/or functional order or disorder is not (thermal) energy order/disorder, and the former is not described by nor directly related to thermodynamic entropy. 

### 3.2. Can the Second Law Be Valid and Entropy Always Increasing in Isolated Systems and the Universe, but (Maybe) Not Necessarily in All Processes and All Space and Time Scales?

The notion that entropy may “decrease” in some open systems or in life processes, or on small space and time scales, and thus violate the Second Law is misleading and inaccurate. Local entropy decrease, due to entropy outflow with heat (thermodynamic entropy is associated with thermal motion, i.e., thermal energy or heat only), should not be confused with impossibility of entropy “reduction by destruction”. Instead of entropy increase and decrease, it would be more appropriate to account for entropy production (or generation) and destruction (the latter impassible). Entropy is generated everywhere and always (and thus overall increased), at any scale without exception (including infinitesimal open systems, micro-fluctuations, gravity or entanglement, far-field interactions), but entropy cannot be destroyed by any means, at any scale, and thus, entropy cannot overall decrease (Box 4).

Box 4 **Entropy Is Always Generated at Any Space and Time Scales and Cannot Be Destroyed by Any Means.**

Entropy is generated everywhere and always (and thus overall increased), at any scale without exception (including life processes, open systems, micro-fluctuations, gravity, or entanglement). Entropy cannot be destroyed by any means, at any scale, and thus, entropy cannot overall decrease. Instead of entropy increase and decrease, it would be more appropriate to account for entropy production (or generation) and destruction (the latter being impossible).

Furthermore, since there is no way to destroy entropy at any scale, it cannot be destroyed locally or temporarily and then be compensated elsewhere later. Non-thermal (ideal, reversible adiabatic) processes are isentropic, but in all real processes, due to all kinds of irreversibilities (friction, mixing, chemical and nuclear reactions, conduction heat transfer or thermal friction, etc.), the work potential is always expended, i.e., dissipated into generated heat, and thus always generating entropy. 

Therefore, entropy is always generated (produced) with heat generation, in limit conserved (in reversible processes), and there is no way to destroy entropy, since during heat conversion to work, entropy is ideally conserved, but it is also generated due to real process irreversibilities.

### 3.3. Can Self-Sustained Non-equilibrium, i.e., Structural Quasi-Equilibrium, Violates the Second Law?

The Second Law had been originally defined for simple compressible substances, allowing heat–work interactions only (where temperature is uniform at equilibrium), and in general, it describes process conditions during spontaneous directional displacement of mass-energy (cyclic or stationary extraction of work), accompanied with irreversible generation (production) of entropy due to partial dissipation of work potential to thermal heat.

The existence and creation of self-sustained, stationary non-equilibrium, i.e., quasi-equilibrium with non-uniform properties (non-uniform temperatures, etc., but without stationary work production) does not destroy entropy, and therefore, does it violate the Second Law (Box 5). Making something perpetually ‘hotter or cooler’ (achieving a temperature difference) does not necessarily imply spontaneous heat transfer from lower to higher temperature, since it can be achieved by adiabatic (ideally isentropic) compression/expansion without heat transfer (e.g., as in refrigeration processes, or ‘vortex tube’), where external work or internal work potential is utilized to create a stationary thermal non-equilibrium, i.e., quasi-equilibrium.

Box 5 **A Quasi-Equilibrium with Non-Uniform Properties Does Not Violate the Second Law.**

The creation and existence of self-sustained, stationary non-equilibrium, i.e., quasi-equilibrium with non-uniform properties (non-uniform temperatures, etc., but without stationary work production) does not destroy entropy; therefore, it does not violate the Second Law.

Dynamic and structural quasi-equilibriums with non-uniform properties, as compared with classical ‘homogeneous equilibrium’, are elusive and may be construed as non-equilibriums, if they are re-structured and in a transient process some useful work is obtained, to allude to violation of the Second Law. However, such work potential is limited to one being stored within such a structure (regardless how small or large) and cannot be utilized as a perpetual (stationary or cyclic) device to continuously generate useful work from within an equilibrium. 

After all, before the *Second Law violation* claims are hypothesized, reliable criteria for the Second Law violation, including proper definition and evaluation of entropy, should be established based on full comprehension of the fundamental laws.

## 4. Other Typical “Entropy and Second Law Misconceptions”

### 4.1. Does Maxwell’s Demon Violate the Second Law?

A demonic being, introduced by Maxwell, to miraculously create thermal non-equilibrium by taking advantage of the non-uniform distribution of molecular velocity in equilibrium, and thereby violate the Second Law of thermodynamics, has been among the most intriguing and elusive wishful concepts for over 150 years now [13,14,15]. Maxwell and his followers focused on “intelligent and effortless gating” of a molecule at a time, but overlooked simultaneous interference of all other thermally-chaotic molecules, while the demon exorcists tried to justify impossible processes with creative but misplaced “compensations” by work of measurements and gate operation, and information storage and memory erasure with entropy generation. It is reasoned phenomenologically and deduced by this author [15] that a Maxwell’s demon operation, against natural forces and without due work effort, is not possible, since it would be against the physics of the chaotic thermal motion, the latter without consistent molecular directional preference for selective timing. Maxwell’s demon (MD) would have miraculous useful effects, but also some catastrophic consequences. The most crucial fact—that the integral, chaotic and simultaneous interactions of all thermal particles on the MD operation—has been overlooked, but focus on a single, opportunistic particle motion is emphasized, as if the other thermal particles would “self-pause to watch” and not interfere during the gate operation. Due effort to suppress such forced interference of other thermal particles would amount to required, major *due work* to establish a macro-non-equilibrium, which is independent and in addition to auxiliary, minor *gate work* of MD to observe molecules and operate a gate (Box 6). The former, thermodynamic due work is unavoidable and substantial, while the latter, MD operational gate work, can be infinitesimally small if the MD operation is perfected to near-reversible actions, and thus making Second Law violation incorrect. After all, the wishful Maxwell’s demon could not had been ever realized and most of the Second Law challenges have been resolved in favor of the Second Law, and never otherwise.

Box 6 **Maxwell’s Demon Requires More Effort Than Just to Operate a Gate [15].**

The most crucial fact—that the integral, chaotic and simultaneous interactions of all thermal particles on the Maxwell Demon’s gate operation—has been overlooked, but focus on a single, opportunistic particle motion is emphasized, as if the other thermal particles would “self-pause to watch” and not interfere during the gate operation. The Maxwell’s demon requires more effort than just to operate a gate.

### 4.2. Can a Vortex Tube and Similar Cooling Devices Violate the Second Law? 

Air injected tangentially into a pipe becomes refrigerated at the center and heated at the periphery, in apparent violation of the Second Law of thermodynamics. Observed in 1933, this remained unexplained for a long time, during which time much debate was produced in the literature. It surprises this author that the observed *vortex tube* stayed unexplained for so long. It only confirms that the Second Law is not fully comprehended by many. 

Even nowadays, the universality of the Second Law is unjustifiably questioned. The physics of the vortex tube should be very clear: it is just another interesting type of refrigeration device that achieves cooling by using the work potential of compressed air. The same compressed air can be used to run a turbine and produce work, to in turn, run a classical refrigeration machine. 

Similar claims of possible Second Law violations or miraculous “thermodynamic magics”, have been unjustifiably hypothesized for many other devices and processes utilizing illusive external or internal work potentials [16].

### 4.3. Could the Second Law Be Violated during Animated Life and Intelligent Processes? 

The Second Law is often challenged in biology, life, and social sciences, including evolution and information sciences—all of which have history rich in confusion. There are other types of “organized structures” in addition to the mass-energy non-equilibrium, such as form and functional design structures, or information/algorithm/template structures, including DNA and RNA structures, with deferent functions and purposes.

Even though the functional form structures and energy structures are similar in some regards and may be described with the same or similar simulation methods, they are not the same and do not have the same physical meaning nor the physical units. Namely, diverse statistical disorders are not the same as thermal-energy disorder, regardless of the fact that they can be described with similar statistical methodology. 

Therefore, thermodynamic entropy [J/K unit] is not the same as information entropy or other entropy, but it is different in terms of physical and logical concepts. Organization/creation of technical (man-made) and natural (including life) structures and thus creation of “local non-equilibrium” is possible and is always happening in many technical and natural processes driven by work potential, using another functional structures (tools, hardware/software templates, information/knowledge and/or diverse intelligent algorithms, DNAs, etc.). 

However, the mass-energy flow within those structures will always and everywhere dissipate work potential (useful energy) and generate entropy (as defined by the Second Law), i.e., at the expense of surrounding/boundary systems’ non-equilibrium or from within structural quasi-equilibrium (Box 7). It may appear that the created non-equilibrium structures are self-organizing from nowhere, from within an equilibrium (and thus violating the Second Law)—due to the lack of proper observations and “accounting” of all mass-energy flows, the latter maybe in “stealth” form or at an undetected rate due to state of our technology and comprehension (as history has shown us many times). Entropy can decrease (locally) but cannot be destroyed anywhere. Miracles remain until we comprehend and explain them!

Box 7 **Creation of Ordered Structures or Live Species Always Generate Thermal Disorder, i.e., Always Generate Entropy.**

It is possible to have water run uphill, heat transported from a colder to hotter body, build a functional (organized) structure, and yes, have natural and life processes create amazing organization and species, but all due to transferred or within work-potential expenditure with dissipation, accompanied with heat and entropy generation, and thus without Second Law violation.

Similarly, we cannot produce cold or hot or life (or any non-equilibrium) from within an equilibrium, without having energy flow from the surroundings, or from within quasi-equilibrium, the latter sometimes may be virtually difficult to observe and measure. Without environmental influence (mass-energy transfer always accompanied with entropy generation), there would be no formation of cyclones, crystals, or life! For example, until a couple of hundred years ago, we did not even know what happens with the energy of a falling stone after it hits the ground, because we could not easily observe or measure its outcome. 

### 4.4. Could the Second Law Be Violated during Micro-Fluctuations? 

Firstly, the time and spatial integrals of micro-quantities have to result in macro-quantities. Therefore, claiming violation of the Second Law on micro-scale or special processes is inappropriate. Secondly, micro-fluctuations make up the macro-equilibrium, do not violate the Second Law, and cannot be “demonized” (without due work) into macro-non-equilibrium (by Maxwell’s demon or otherwise)(Box 8). 

Furthermore, on microscopic and quantum scales, the macroscopic quantities cannot be defined as such, but only as quasi-equivalents for certain comparisons, without true meanings. The underlying mass-energy structures and processes at the utmost micro-scales are more complex and undetected at our present state of tooling and intellectual comprehension. However, their integral manifestations at the macroscopic level are more realistically observable and reliable, and thus being the *check-and-balance* of microscopic and quantum hypotheses.

Since true reversibility is present in ideal equilibrium processes—but for real processes (process means directional forcing of displacement of mass-energy fluxes), at least an infinitesimal non-equilibrium forcing is necessary to provide direction—then all real processes should be at least infinitesimally irreversible, and thus all real processes are irreversible, with reversibility being an ideal limiting case (as is the equilibrium). Since the macroscopic processes are integral outcomes of micro- and quantum processes, then they also should be at least infinitesimally irreversible, even if non-observable at our present state of tooling and comprehension. Reversibility and equilibrium are only idealizations, as are many other concepts.

Box 8 **Near-Reversible Micro-Fluctuations Make Up Macro-Equilibrium and Cannot Be Demonized to Destroy Entropy.**

Thermal micro-fluctuations do not represent increase and decrease in macro-thermal properties as micro-violation of the Second Law, but to the contrary, the thermal micro-fluctuations represent the very existence of self-sustained, thermal macro-equilibrium with maximum entropy, i.e., minimum Boltzmann’s *H*-value quantity.

Thermal phenomena, temperature and entropy are macroscopic average quantities of the random distribution of fluctuating micro-thermal properties, including self-sustained equilibrium processes (macroscopic quantities constant in equilibrium, the essence of *thermal equilibrium*, made up of randomized and self-sustained, non-uniform micro-motions). For example, the Maxwell–Boltzmann thermal distribution within an ideal gas in equilibrium, is a non-uniform spatial and temporal distribution of micro-properties of the molecules’ positions and momenta. Therefore, such thermal micro-fluctuations do not represent an increase and decrease in macro-thermal properties as micro-violation of the Second Law, but to the contrary, the thermal micro-fluctuations represent the very existence of self-sustained, thermal macro-equilibrium with maximum entropy, i.e., minimum Boltzmann’s *H*-value quantity. 

Fluctuating phenomena in perfect equilibrium are reversible, and thus isentropic, so that any reduction in entropy reported in the literature (whatever that means), may be due to ‘incomplete’ entropy definitions at the micro- and submicro-scales, approximate accounting for thermal and/or overlooking diverse displacement contributions to entropy. For example, during isothermal free expansion, entropy is generated due to volume displacement, and similarly could be due to subtle and illusive micro- and submicro-‘displacements’, including particle ‘correlations’ and/or quantum entanglements in respective force fields, etc. Further, note that fluctuating temperature does not always mean fluctuation of entropy, as demonstrated by the well-known isentropic compression expansion, for example [15]. Therefore, fluctuation phenomena do not violate the Second Law, since the temperature fluctuations could be adiabatic (in limit, isentropic) or due to heat transfer fluctuations.

### 4.5. Could the Second Law Be Violated Locally and Temporarily and “Compensated” Elsewhere Later?

Local and temporal destruction of entropy is impossible (as already reasoned) and cannot be “compensated” elsewhere or at a later time, to “post-satisfy” the Second Law. “Entropy of an isolated, closed system (or universe) is always increasing” is a necessary but not sufficient local condition of the Second Law of thermodynamics: entropy cannot be destroyed, locally or at a time, and *compensated* by generation elsewhere or later (Box 9). It would be equivalent to allow rivers to “spontaneously flow” uphill and compensate it by more downhill flow elsewhere or later. Entropy is generated everywhere and always, at any scale without exception, and cannot be destroyed by any means at any scale (as is reasoned above).

Box 9 **Entropy Cannot Be Destroyed, Locally or at a Time, and “Compensated” by Generation Elsewhere or Later.**

Entropy is generated everywhere and always, at any scale without exception, and cannot be destroyed by any means at any scale. “Entropy of an isolated, closed system (or universe) is always increasing” is a necessary but not sufficient condition of the Second Law of thermodynamics.

*Impossibility of entropy reduction by destruction should not be confused with local entropy decrease due to entropy outflow with heat.*

### 4.6. Thermodynamic “Arrow of Time”

Arrow of time is supposed to be a general concept independent from a clock design or “personal perception”, in the way thermodynamic temperature is independent of a thermometer design, or how light speed is independent of observer’s speed. The time and entropy are always being irreversibly generated and overall irreversibly increased. It is premature to make any definitive relationship between the two before their correlation is well established. 

However, the *thermodynamic arrow of time*, as general irreversibility, i.e., the directionality of all processes with entropy generation without exception, may be the answer to “*where does our arrow of time come from*?” Even if a reversible arrow of time could go backwards (reversibility of *time arrow*), it will not be violation of the Second Law, but it would be a limiting ideal, reversible case (entropy would have been conserved, not destroyed) (Box 10).

Box 10 **The “Thermodynamic Arrow of Time” May Be the Answer to *“Where Does Our Arrow of Time Come From?”***

Since all real processes in nature are at least infinitesimally irreversible, reversibility being the limiting concept, then the real time could be slowed down, in limit stopped, but not reversed.

And many other hypothetical and unjustified claims of Second Law anomalies or violations are never ending. However, no Second Law challenge has ever been proven to violate the Second Law but to the contrary.

## 5. Further Comments

To summarize again: All processes in nature (*cause-and-effect* phenomena) are irreversibly driven in a forced direction by non-equilibrium useful energy (work potential), where mass-energy flux is transferred while conserved (the *First Law*), but in part (and ultimately in-whole at equilibrium), the work potential is dissipated to thermal heat, and thus always and irreversibly generating entropy (the *Second Law*).

Claims by the Second Law challengers, that with creative devices, “*challengers’ demons*”, it is possible to embed them into an equilibrium environment and extract perpetual ‘*useful* work’ are philosophically and scientifically unsound. Such a magic and wishful ‘*demon*’, if possible and embedded as a ‘black box’ in a system or environment at equilibrium, to produce a spontaneous, steady-state (stationary or cyclic) work-extraction process from within such an equilibrium, would also be a ‘catastrophically unstable’ process, with the potential to ‘syphon’ all existing mass-energy in an infinitesimal-size singularity with infinite mass-energy potential, a ‘*monster black energy hole’*. If it were ever possible, we would not exist ‘as we know it,’ here and now!

Box 11 **Hypotheses of Second Law Violation Are Still to Be Proven.**

All resolved paradoxes and misleading violations of the Second Law, to date, have been resolved in favor of the Second Law and never against. We are still to witness a single, still open ‘Second Law violation’, to be confirmed.

The challengers misinterpret the fundamental laws, present elusive hypotheses, and perform incomplete and misleading, biased experiments, always short of straightforward confirmation of their Second Law violation claims. That is why all resolved, challengers’ paradoxes and misleading violations of the Second Law, to date, have been resolved in favor of the Second Law and never against (Box 11). We are still to witness a single, still open *‘Second Law violation’*, to be confirmed.

In summary, the current frenzy about the Second Law violation (a ‘*perpetual motion of the second kind*’, obtaining *useful energy* from within equilibrium) is in many ways similar to the prior frenzy about the ‘*First Law violation*’ (obtaining *energy* from nowhere). It is hard to believe that a serious scientist nowadays, who truly comprehends the Second Law and its essence, would challenge it based on incomplete and elusive facts [17].
